# General Intelligence

**Published:** 1844-11

**Authors:** 


### GENERAL INTELLIGENCE.

### CIRCULAR TO PHYSICIANS.

At the semi-annual meeting of the Erie Co. Medical Society, held June 11th, a committee of five, consisting of Drs. Sprague and Flint of Buffalo, Pratt of Evans, Wallace of Aurora, and M'Beth of Wales, was appointed to investigate in as far as practicable, and especially from facts occurring in this county, the causes, history, pathology and best method of treatment of Puerperal fever, and report at the next meeting of the Society. By amendment to the motion, Epidemic Erysipelas was also included.

The committee are desirous to fulfil as far as possible the trust committed to them, and in order to do so they wish to secure the co-operation of Physicians generally. They hope that sufficient interest will be felt in the subject and undertaking, for every member of the profession whom this circular may reach, to furnish the committee with such facts as have already fallen, and may hereafter occur under their observation, together with the views and practical conclusions which they have deduced from their own experience.

For the sake of uniformity, and for mutual convenience, a list is added of the several points concerning which in each case, precise and full information is solicited. It is desired that the cases will be recorded separately, embracing facts relating to the several points included in the following list, and in the same order.

Of the cases which have already occurred under your observation, please note the details according to your best recollection, if no record was made at the time of their occurrence. In those which may hereafter occur, the records will of course, be more reliable if they are noted simultaneously with the progress of the cases, or, at least, immediately after their termination. It will be important to distinguish the cases wherein the facts are given from recollection, from those where they are noted down from immediate observation.

Any views or suggestions accompanying the cases will be acceptable, and duly acknowledged, as also any considerations which may appear to have a bearing, however remote, on the subject.

Communications, *if post paid*, will be received by the editor of the *Illinois Medical and Surgical Journal*, up to the latter part of December next, and forwarded by him to the committee. Should a large number of cases with the details fully recorded in accordance with the plan adopted be received, it is believed that their careful analysis and comparison will lead to very interesting and important results. AUSTIN FLINT,

*Chairman of Committee.*

---

The following is a list of the several points concerning which information is requested of the facts occurring in each case separately. By recording the details in the same order, whenever a case presents itself, the labor will be slight.

Date, name of patient, age, number of children, occupation and habits, general health and constitution, state of health during gestation. Had the Physician shortly previous to the accouchment, visited patient or patients with the puerperal fever or erysipelas? Was the labor natural, or were any unusual circumstances attending it? Of the lochial discharge. Of the secretion of milk, did it take place, and was it scanty or abundant? Date of the attack from the time of labor. Did it come on suddenly, or did the symptoms supervene gradually? Of chills and rigors. Of pain, its location, degree and character. Of abdominal tenderness. Of tumefaction of the abdomen. Of the alvine discharges. Were cathartics administered before the attack, and of what did they consist? Of the pulse, its frequency, size, hardness or softness, compressibility, &c. Of the skin, hot or cool, dry or moist, perspirable, &c. Of the tongue. Of the mind previous to and after the attack. Did the patient anticipate, and dread the disease? Thirst, nausea, vomiting, or other gastric symptoms. Of the urinary secretions, abundant or scanty, appearance of urine. Of the muscular strength, prostration or exhaustion, degree and character. What mode of treatment was pursued, and the immediate effects? What appearance did the blood present, if blood-letting was practised? Duration of the disease. Termination in death or recovery. Post mortem appearances. General remarks. What other diseases have prevailed simultaneously, and have all diseases been marked by any character or characters peculiar to the season? Did epidemic erysipelas prevail before or during the cases with puerperal fever, and what were the prominent characters appertaining to this disease. Detailed records of cases of erysipelas will be very acceptable.

[We call the attention of our readers to the circular above, and hope that the desire of the committee may meet with the prompt attention the importance of the subject demands. Should the contributions sent by our readers be numerous and valuable, we hope to be able to lay before them in the report of the committee, results important to the profession, and creditable to the contributors.—Ed.]

